# *Fit*EM2EM—Tools for Low Resolution Study of Macromolecular Assembly and Dynamics

**DOI:** 10.1371/journal.pone.0003594

**Published:** 2008-10-31

**Authors:** Ziv Frankenstein, Joseph Sperling, Ruth Sperling, Miriam Eisenstein

**Affiliations:** 1 Department of Structural Biology, Weizmann Institute of Science, Rehovot, Israel; 2 Department of Organic Chemistry, Weizmann Institute of Science, Rehovot, Israel; 3 Department of Genetics, The Hebrew University of Jerusalem, Jerusalem, Israel; 4 Department of Chemical Research Support, Weizmann Institute of Science, Rehovot, Israel; University of Reading, United Kingdom

## Abstract

Studies of the structure and dynamics of macromolecular assemblies often involve comparison of low resolution models obtained using different techniques such as electron microscopy or atomic force microscopy. We present new computational tools for comparing (matching) and docking of low resolution structures, based on shape complementarity. The matched or docked objects are represented by three dimensional grids where the value of each grid point depends on its position with regard to the interior, surface or exterior of the object. The grids are correlated using fast Fourier transformations producing either matches of related objects or docking models depending on the details of the grid representations. The procedures incorporate thickening and smoothing of the surfaces of the objects which effectively compensates for differences in the resolution of the matched/docked objects, circumventing the need for resolution modification. The presented matching tool *Fit*EM2EMin successfully fitted electron microscopy structures obtained at different resolutions, different conformers of the same structure and partial structures, ranking correct matches at the top in every case. The differences between the grid representations of the matched objects can be used to study conformation differences or to characterize the size and shape of substructures. The presented low-to-low docking tool *Fit*EM2EMout ranked the expected models at the top.

## Introduction

The structures of large and flexible macromolecules and macromolecular assemblies are hard to determine and the current experimental techniques often produce only medium to low resolution models. Single particle electron microscopy (EM) is one such technique that produces models at resolutions as high as 4 Å [1], [2]. Other techniques that produce low resolution structures are atomic force microscopy (AFM) [3], [4] and small angle X-ray scattering (SAXS) [5], [6]. Recently, single particle EM in vitrified ice (cryo-EM) was used to study the structural heterogeneity of macromolecules and assemblies, exploiting the fact that the fast vitrification traps samples at different conformations and different stages of activity [7]–[9]. Characterizing and analyzing structural heterogeneity of macromolecules is challenging as it requires comparison of low resolution models obtained at different resolutions and sometimes using different techniques, each carrying its typical experimental error. Currently such comparisons are customarily done by manual fitting of the models (e.g. [10]–[13]). Similarly, manual fitting is commonly used in assembly modeling studies (e.g. [14]–[16]).

Many programs that match atomic structures to EM maps have been previously presented (reviewed in [17]) however these tools were not tested for matching of one EM map to another. A computerized tool for comparing low resolution 3D models is evidently necessary. It is however a non-trivial task because maps at resolutions lower than 15 Å do not carry enough features for density comparison [18]–[20]; in addition experimental error, density cutoff uncertainty and resolution differences must by considered [18], [21]. Recently it was shown that the combination of shape matching with maximization of density overlap provides a superior tool for matching atomic resolution models to EM maps in the resolution range of 12–25 Å (high-to-low resolution matching) [22]. We present here new tools designed for comparing and matching low resolution models (*Fit*EM2EMin for low-to-low resolution matching) and for docking of such models (*Fit*EM2EMout). The tools are based on the shape complementarity algorithm, MolFit [23]. Hence, we employ three dimensional (3D) grid representations of the low resolution objects and use fast Fourier transformations (FFT) to match or dock them. We tested *Fit*EM2EMin on ten pairs of related EM maps and found that it can efficiently match EM maps obtained at different resolutions and using different EM techniques. Moreover, models of different conformers were successfully matched and partial structures were fitted into larger assemblies. The difference between the grid representations of the matched models can be used to study conformation changes or to predict the shape and size of substructures. Docking of low resolution EM maps with *Fit*EM2EMout identified the expected positions and ranked them at the top of the list of predicted models.

## Results

The matching and docking tools *Fit*EM2EMin and *Fit*EM2EMout test many relative orientations of the matched/docked objects. The EM maps were therefore represented as ensembles of spheres (“virtual atoms”) that can be easily rotated and translated. The spheres fill the objects' envelopes at the selected electron density cutoff values. The representation of the EM objects is governed by the parameter *w*, which determines the extension of the surface layer of the object and its outer shape. This parameter is likely to be affected by the resolution of the EM map and the density cutoff; its optimization was executed by matching several EM structures, each to its copy (self-matching). We chose to match symmetrical or pseudo-symmetrical EM structures expecting several correct matches (the actual number depends on the symmetry) some of which are identity matches and other are approximate either because the symmetry of the structure is not perfect or because the symmetry operation does not comply with the rotational step employed in the scan and only nearby orientations are sampled.

### Self-matching of EM maps

Nine EM structures determined at different resolutions (from 11.2 to 42.2 Å) and obtained using different experimental procedures were selected for this test (see Table 1). EM maps were taken from the Macromolecular Structure Database (EMDB) at http://www.ebi.ac.uk/msd-srv/emsearch. The electron density cutoffs were obtained from the investigators in most cases; in other cases the cutoffs were manually approximated to reproduce the published EM objects.

**Table 1:** Benchmark of EM structures used in the optimization of *w*, the parameter that determines the width and shape of the surface layer of object A.

System	EMDB code	EM method/complex symmetry	Resolution^a^ (Å)	Optimal *w* range	R1^b^	R2^c^
ATP-*T*ClpB mutant [10]	1244	cryo-EM/C_6_	11.2	3.2–3.6	9.4	8.6
TET1 peptidase [42]	1188	cryo–EM/tetrahedral 12-mer	14	2.6–3.0	10.4	10.6
apo-*T*ClpB [10]	1241	cryo-EM/C_6_	17.7	3.0–3.4	9.2	8.0
TIP48/TIP49 complex [43]	1317	uranyl acetate negative staining/C_6_	20	2.0–2.2	9.9	11.3
GroES-ADP_7_-GroEL-ATP_7_ [27]	1046	cryo EM/C_7_	23.5	1.8–2.2	8.1	9.0
MCM helicase [44]	1134	uranyl acetate negative staining/C_6_	25	1.8–2.2	10.1	10.9
SV40 T antigen [45]	1024	uranyl acetate negative staining/C_6_	29	1.8	10.3	11.0
DnaB [25]	1022	cryo-EM/C_3_	34.5	1.6–2.0	10.3	9.7
DnaB.DnaC complex [25]	1023	cryo-EM/C_3_	42.2	1.4	8.8	7.9
DnaB.DnaC complex at two resolutions [25], [26]	1023/1017	cryo-EM/C_3_	34.1	1.8–2.0	10.4	9.5
GroES-ADP_7_-GroEL-ATP_7_ and GroES-ATP_7_-GroEL [27], [28]	1046/1180	cryo EM/C_7_	15.6	2.2–2.6	6.7	7.8
ATP-*T*ClpB mutant and ADP-*T*ClpB [10]	1244/1242	cryo-EM/C_6_	14.0	2.6–3.0	8.2	8.4
AMPPNP-*T*ClpB and ADP-*T*ClpB [10]	1243/1242	cryo-EM/C_6_	14.4	3.0–3.4	9.4	8.4
GroES-ADP_7_-GroEL-ATP_7_ and GroEL-ATP_7_ [27]	1046/1047	cryo-EM/C_7_	19.2	2.6–3.0	8.4	8.1

a The resolution quoted here is the value in the EMDB; average resolution is given for the pairs of related structures.

b R1 is the virtual atoms radius calculated with the central values of the *w* ranges in column 5.

c R2 is the virtual atoms radius calculated with *w* values derived from the linear dependency of *w* on the resolution (Figure 1).

Several rotation-translation matching scans were preformed for each structure in the benchmark, employing different *w* values. The rotations interval was fixed at 12° in these tests. The highest score in each scan was either that of the identity match (no rotation; translation within 3 grid steps of the expected position) or of an exact symmetry related match. Matches that deviate by ∼6° (half the rotation step) from a correct position (identity or symmetry related) were ranked after the exact symmetry matches and were followed by matches that deviate by ∼12° (the rotations grid interval). In only one case, the SV40 T antigen, a false match, rotated by 180° about an axis perpendicular to the C_6_ symmetry axis, was ranked among the matches with ∼12° deviation; this is a false match because the SV40 T antigen does not have D_6_ symmetry even at the low resolution of 29 Å. The score of this false match is however only 54% of the score of the top ranked correct match.

The optimal *w* for each map was chosen based on 2 criteria: the first was good tolerance of deviations up to 6° from a correct position, thus the scores of such matches were close to the score of the identity match (≥80%). The second criterion was that deviations of 12° were tolerated yet were distinct from the correct matches; hence the scores of these matches were positive but dropped to ∼15%–70% of the score of the identity match. It appears that ranges of *w* comply with these criteria for each system (see Table 2), which is indicative of only moderate sensitivity of the matching results to *w*. However, the *w* ranges vary considerably between systems and *w* is negatively correlated with the EM map grid interval (R^2^ = 0.85 for the central values of the *w* ranges) and with the resolution (R^2^ = 0.78; see Figure 1). This is expected, because the optimal *w* were chosen to confer limited tolerance of structural mismatches the extent of which depends on the experimental resolution and the grid interval. In contrast, *w* is not correlated with the density cutoff (R^2^ = 0.16). This is also expected, because the density cutoff used to produce the molecular envelope depends on the molecular volume and on the background scattering of the electrons beam hence on the EM method and the experimental conditions [21], [24].

**Figure 1:**
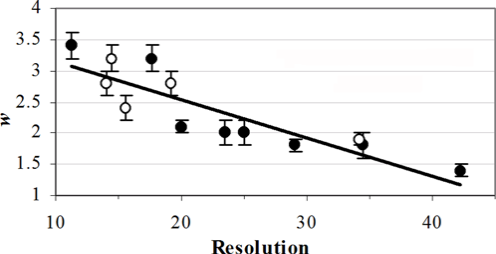
The dependency of the optimal *w* values on the resolution of the matched EM maps. The full circles depict the results of self-matching (for systems listed in Table 1). The hollow circles depict the results of matching of related EM maps (the average resolution is used here). The regression line (y = −0.0612x+3.7551; R^2^ = 0.78) is based only on the full circles. The error bars reflect the range of the optimal *w*.

**Table 2:** Matching related EM maps.

System^a^	EMDB code and resolution (Å)	*w* and *g*	No. of matches and correct matches with score>−10000	Rank of highest ranking false match
DnaB.DnaC complex [25]	1023, 42.2	1.7	277	39
DnaB.DnaC complex [26]	1017, 26.0	3.75	38	
GroES-ADP_7_-GroEL-ATP_7_ [27]	1046, 23.5	2.8	57	24
GroES-ATP_7_-GroEL [28]	1180, 7.7	2.11	27	
GroES-ADP_7_-GroEL-ATP_7_ [27]	1046, 23.5	2.6	21	17
GroEL-ATP_7_ [27]	1047, 14.9	2.35	18	
GroES-ATP_7_-GroEL [28]	1180, 7.7	3.2	915	20
GroES-ATP_7_-(single-ring)GroEL [29]	1286, 10.4	1.79	29	
ATP-*T*ClpB mutant [10]	1244, 11.2	2.9	145	66
ADP-*T*ClpB [10]	1242, 16.7	2.22	89	
AMPPNP-*T*ClpB [10]	1243, 12.1	2.9	84	73
ADP-*T*ClpB [10]	1242, 16.7	2.25	72	
AMPPNP-*T*ClpB [10]	1243, 12.1	3.0	12	3
ATP-*T*ClpB mutant [10]	1244, 11.2	2.22	12	
TFIID-closed [30] b	1194, 33.0	1.7	1	13
TFIID-open [30]	1196, 35.0	5.12	1	
apo-ORC [31] b	1252, 34.0	1.7	39	14
ATP-ORC [31]	1253, 34.0	5.26	13	
Ribosome 80S [32] b	1093, 18.3	2.4	262	3
Ribosome 40S [32]	1092, 25.3	3.69	3	

a The first structure in each pair is the stationary object A and the second structure is the moving object B.

b Values are given for matching with the lowest density cutoff for object A and highest cutoff for object B (see text).

Unlike *w*, the virtual atoms radii (determined by *w* and *g*, see Methods) are not correlated with the resolution (see Table 2). The average virtual atoms radius calculated with the central values of the *w* ranges is 9.6 Å±0.8. The corresponding average calculated with *w* derived from the dependency graph in Figure 1 is 9.7 Å±1.3. It appears that the optimal *w* values modify the resolution of the EM map creating surfaces that are not resolution dependent. Virtual atoms with radius of 9.7 Å produce a surface that confers tolerance to small structural mismatches or to mis-positioning due to the stepwise scanning of the rotation-translation space. This result is very important in view of the wide resolution range of EM structures.

The search volume in these simple tests, in which objects A and B are identical, consists of the outer part of the surface layer of object A, corresponding to the difference between the surface extensions applied to the two objects. The thickness of this layer, (*w*−1)*g*, depends on the EM map resolution. Therefore, self-matching of the higher resolution EM maps, which often are presented at small grid intervals, leads to a shift of the highest scoring model at the identity orientation from the expected position (≤3 grid steps). The best matching results are obtained when *w*>1 thus allowing some freedom for object B to move and adjust to object A.

### Matching related EM maps

A more realistic test of the *Fit*EM2EMin algorithm was the matching of related EM maps, including matching of the same object determined at different resolutions, matching of partial structures and matching of different conformers. Optimal *w* ranges were reevaluated for five pairs of structures (see Table 1). Rotation-translation scans were performed employing several *w* values and ranges of optimal *w* were determined using the abovementioned criteria. The resultant *w* ranges for these five cases fit reasonably well to the regression line of *w* versus resolution obtained in the self-matching of EM maps, as shown in Figure 1.

**Figure 2:**
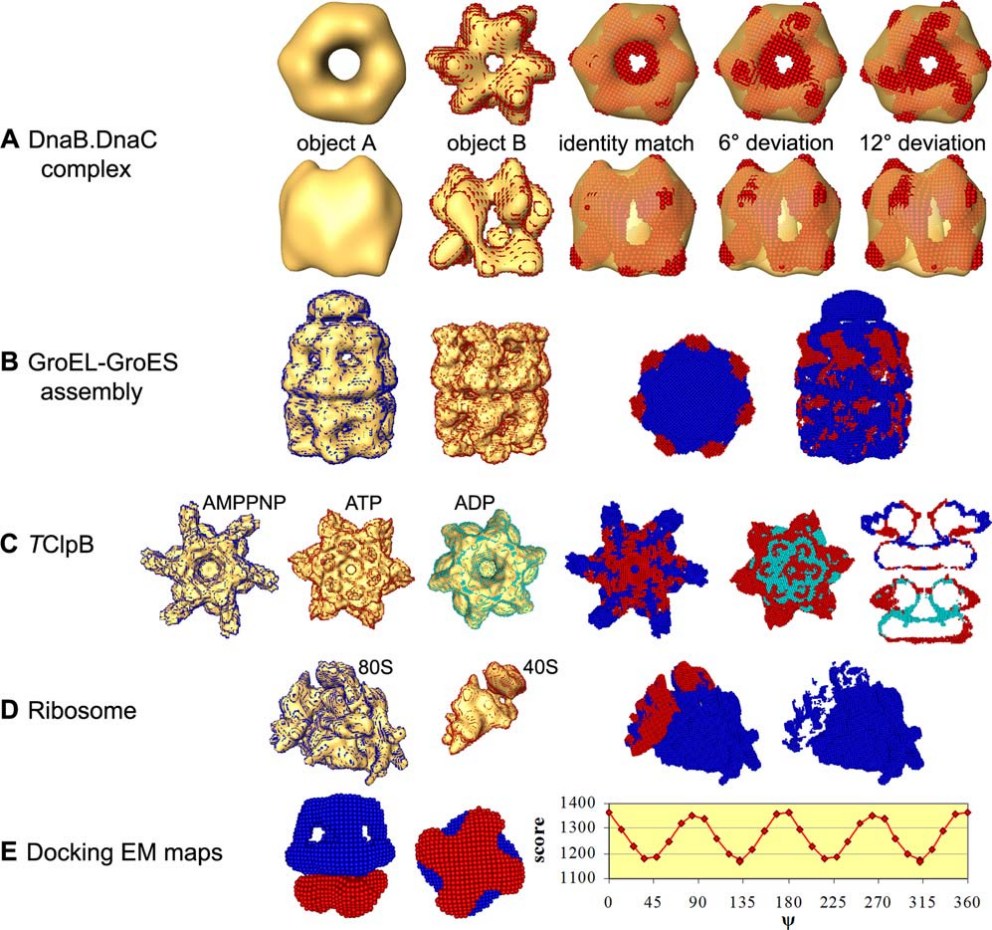
Examples of low-to-low resolution matching and docking. The top ranking matches are shown, obtained in *Fit*EM2EMin scans that employ *w* values calculated from the dependency graph in Figure 1. EM envelopes are shown in yellow. The DifferenceGrid portions where object A protrudes out of object B are colored as object A, and vise versa for object B. The individual images were prepared with the software package Amira and are not to scale. Details for each row of pictures are listed from left to right. (A) Matching of two EM structures of the DnaB.DnaC complex (top and side views). Shown are the EM envelope of objects A (resolution 42.2 Å); the virtual atoms representation of object B (resolution 26 Å) in red within its EM envelope; the identity match (score 1963, ranked 1); match deviating by 6° (score 1352, ranked 4); match deviating by 12° (score 96, ranked 7). (B) The virtual atoms representations of GroES-ADP_7_-GroEL-ATP_7_ (blue) and GroEL-ATP_7_ (red) within their EM envelopes; top and side views of the DifferenceGrid results. (C) Virtual atoms representations of AMPPNP-*T*ClpB (blue), ATP-*T*ClpB mutant (red) and ADP-*T*ClpB (cyan); top views of the DifferenceGrid results for the match of AMPPNP-*T*ClpB to ATP-*T*ClpB mutant and for the match of ATP-*T*ClpB mutant to ADP-*T*ClpB; sections through the side views of the DifferenceGrid results for the same matches. (D) Virtual atoms representations of the 80S ribosome (blue) and the 40S subunit (red); the top ranking match and the DifferenceGrid results. (E) Side and bottom views of the top ranking docking model between Kv4.2*-KChlP2 (blue) and the simulated map of β2_4_ (red); changes in the complementarity score as function of ψ, the rotation angle about the 4-fold axis of the predicted complex.

Ten pairs of related EM maps were used to test our matching algorithm and to demonstrate its performance. In one case, the EM maps of the complex between helicase DnaB and its DnaC loading partner, determined at very different resolutions (26 and 42.2 Å) were matched [25], [26]. Despite the large difference in resolution our algorithm ranked a correct match at the top. A false match with a negative score is ranked 39 (see Table 2). Figure 2A presents the correct top ranking match and additional matches deviating by 6° or 12°.

The GroEL/GroES chaperonin complex is a molecular machine whose components assembly and disassembly upon ATP binding and hydrolysis is accompanied by conformation changes. GroEL consists of two 7-subunits rings, which can be in the apo state or loaded with either ATP or ADP; GroES consists of a single 7-subunits ring. The structures of the complexes GroES-ADP_7_-GroEL-ATP_7_ [27] and GroES-ATP_7_-GroEL [28], which were determined at very different resolutions (23.5 and 7.7 Å), are expected to be similar [28]. Our algorithm matched these objects correctly, ranking a correct match at the top. We then matched GroEL-ATP_7_ [27] to GroES-ADP_7_-GroEL-ATP_7_ [27] and the GroES-ATP_7_-(single-ring)GroEL [29] to GroES-ATP_7_-GroEL [28]. These complexes were determined at different resolutions (see Table 2) and have different compositions; the first object in each pair is a substructure of the second. In both tests correct matches were ranked at the top despite the different assembly sizes and differences in conformation. The DifferenceGrid results for the GroEL-ATP_7_ and GroES-ADP_7_-GroEL-ATP_7_ pair highlight the conformation differences upon ATP binding to the GroEL *cis* ring (see Figure 2B) whereas the results for the GroES-ATP_7_-GroEL and GroES-ATP_7_-(single-ring)GroEL pair suggest that these two complexes are similar.

The matching of the different conformers of *T*ClpB provided another test for the influence of conformation changes on the success rate of our procedure. *T*ClpB is an ATP-dependant molecular chaperone which disaggregates stress-damaged proteins. Its structure consists of two hexameric rings (AAA-1 and AAA-2) rotated by 23° about the co-axial 6-fold rotation axis. It was suggested that the exposed face of the AAA-1 ring binds the substrate which is then threaded through the central pore [10]. The rings are of similar size in the ATP bound *T*ClpB mutant (E271A, E668A) but not in apo *T*ClpB. We note that in the self-matching of the ATP bound complex our algorithm identified a pseudo-symmetric match in which the rings interchange (rotation of 180° about an in-plane axis plus 24° about the 6-fold axis). The score of this (false) match is however only 22% of the score of the correct match. The self-docking of apo *T*ClpB did not produce such a pseudo-symmetrical model.

We matched the ATP bound *T*ClpB mutant and the AMPPNP and ADP bound hexamers to each other. In all three cases correct matches, similar to the manual fittings [10], were ranked at the top. The ATP- and ADP-bound *T*ClpB matching was executed employing rotational intervals of 8, 12 or 16°, in order to test the sensitivity of the results to this parameter. A correct match was ranked at the top in every case; the scores of the top ranking models obtained in the 8° and 16° searches were slightly higher than the score obtained in the 12° search, indicating that a relative rotation of 4° marginally improves the match. This result indicates that the 12° rotation interval is adequate for identifying the correct match; yet, top ranking matches should perhaps be refined by testing small local rotations from the predicted position.

The difference between the grid representations of AMPPNP-*T*clpB and the top ranking matched ATP-*T*ClpB, shown in Figure 2C, is in line with the observations of Lee et al. [10]. It highlights the extended spokes in the *T*ClpB-AMPPNP complex compared to the shorter and thicker spokes in ATP-*T*clpB. There is additional density on top of the upper ring in the ATP-*T*ClpB mutant complex and in the central pore region, making the pore narrower. The differences in the shapes of the lower ring of the two objects are minor and may be related to errors in the EM maps and in the matching. The radial spokes are even shorter in the ADP-*T*clpB complex and the central pore of the upper ring is narrower, in particular in the lower half. We note additional structural differences at the interface between the rings.

Two additional examples for matching of different conformers of the same molecule are the closed and open structures of the human transcription factor TFIID [30] and the apo and ATP bound Drosopila origin recognition complex (ORC) [31]. In these cases we also tested the effect of small changes in the density cutoff of the EM map on the matching results. We therefore gradually increased the volume of object A (by slightly decreasing the density cutoff value) and decreased the volume of object B (by increasing the density cutoff value). A correct match was ranked at the top in all the scans however the complementarity appeared to be better (higher scores) as the density cutoff of object A was reduced and that of object B was increased. Notably, in both examples the EM map grid intervals and consequently the grid interval used in the *Fit*EM2EMin matching, are particularly large (>5 Å) but the identification of correct matches was not affected.

Finally we matched the 40S subunit of the human ribosome [32] to the whole ribosome (80S) assembly formed by the 40S and 60S subunits. The effect of the density cutoff change in this case was the same as described above. Thus the position of the 40S subunit within the 80S structure in the top ranked match did not change. The complementarity score however gradually increased as the density cutoff value for the 80S structure was decreased and that of the 40S structure was slightly increased, indicating that the shape and size of the 40S subunit match better within the 80S ribosome. The DifferenceGrid results (see Figure 2D) for the top ranking match can be used to approximate the shape and size of the ribosomal 60S subunit, producing a similar result to the subunits separation presented by Sphan et al. [32].

### Docking EM maps

Docking of low resolution objects is important in assembly modeling and the program *Fit*EM2EMout was designed to perform such tasks. We could not find EM maps of substructures whose overall structure is known in order to calibrate the parameters of *Fit*EM2EMout and test the program. Therefore, the same parameter values and grid representations as described for *Fit*EM2EMin were employed and simulated electron density maps were docked to EM maps. The simulated maps were calculated using the pdb2vol and map2map modules of *Situs* [19]. The electron density cutoff for the simulated map was chosen to encompass all the atoms in the corresponding PDB structure.

In the first test, the coordinates of the *trans* ring of GroEL [33] from PDB [34] entry 1AON were used to calculate an electron density map at 10 Å resolution (the resolution of the docking partner). This map was docked to the EM map of GroES-ATP_7_-(single-ring)GroEL complex [29]. Several symmetry related correct matches were ranked at the top followed by a false match (ranked 5) in which the tip of GroES makes contact with the central void of the GroEL *trans* ring. Fitting of an extreme value distribution function to the distribution of scores [35] provided estimated *E* values of 5.6×10^−4^ and 2.1×10^−3^, respectively, for the highest ranking correct and false models.

The second test involved docking of a simulated map of the tetramer of the oxidoreductase β2 subunit [36] (PDB entry 1EXB) to the EM map of the 4∶4 complex between the pore-forming Kv4.2 subunits and the regulatory KChlP2 subunits [37]. The resolutions of both the EM map and the simulated electron density map were 21 Å. The β2 surface that faces the T1 domain of the pore forming subunit in the T1_4_-β2_4_ complex [36] is expected to make contact with Kv4.2*-KChlP2 [37]; indeed this general position of the simulated map of β2_4_ is observed in many high ranking docking models. These models differ in the rotation angle of the β2 tetramer about the 4-fold symmetry axis and the docking scores change periodically as function of this angle (see Figure 2E). The score difference between the best and worst positions is within 3σ. The highest scoring false model, in which the symmetry axis of β2_4_ is not aligned with that of Kv4.2*-KChlP2, is ranked 35. The *E* values for the highest ranking correct and false models are 5.12×10^−6^ and 1.39×10^−3^, respectively.

## Discussion

The recent upsurge of low resolution structures obtained by different experimental techniques and the capability to study global structural changes by cryo electron microscopy, introduced a need for computational methods to match, compare and dock such structures. We present here tools designed for low-to-low resolution matching and docking. By extending and smoothing of the surfaces of the objects our matching procedure *Fit*EM2EMin becomes resolution independent allowing comparison or fitting of objects obtained at very different resolutions without resorting to resolution modification. Such modification (that is often used in fitting of atomic structures into EM maps) requires knowledge of the target resolution, the experimental determination of which is susceptible to inaccuracies, especially in low resolution studies [38]. We show that *Fit*EM2EMin can match different conformers and fit a partial structure into a larger assembly. The procedure is not sensitive to the exact values of the parameters and ranges of *w* confer identification of correct and nearly correct matches and distinction from false matches. Similarly, small changes in the EM maps density cutoff affect the complementarity score but not the relative position of the matched objects or the rank of the correct match.

The success rate of *Fit*EM2EMin is excellent. Thus, in every case correct matches are obtained at the top. We attribute this success to the limited search volume (the interior plus surface of object A), which allows only few matches with positive scores. When substructures are matched into the whole structure the search volume increases considerably and the results become more similar to docking results. Thus, the number of false models obtained in the partial matching tests 80S/40S and GroES-ATP7-GroEL/GroES-ATP_7_-(single-ring)GroEL is higher than in most other tested cases (see Table 2). Nevertheless the difference between the scores of the correct match (top rank) and the highest ranked false match is large, facilitating distinction between them. The DifferenceGrid tool highlights major structural differences between the matched objects. In the partial docking of the 40S ribosomal subunit into the 80S ribosome this tool can be used to predict the size and shape of the 60S ribosomal subunit.

Docking is expected to produce weaker distinction between correct and false models than matching because the search space is much less limited. Nevertheless, our procedure *Fit*EM2EMout ranked the expected docking models at the top with considerable score gap from that of the highest ranked false models.

The procedures presented here can also be used for matching of atomic resolution structures to EM maps because the large grid effectively lowers the resolution of the atomic representation of an object. In this study the electron density values were used only for determining the shape of the matched or docked objects. These values can however be used to guide the matching or docking, for example by requiring that high density regions, that may represent RNA or DNA regions, overlap. The guiding can be achieved in a similar manner to the weighting procedure previously described by our group [39]. Notably, such guided matching or docking can use experimental data regarding the positions of substructures or specific interactions, from different sources.

## Methods

### Converting EM maps into easily rotateable 3D objects

EM maps are given as 3D grids of electron density values and the objects are visualized by selecting one or more density cutoffs and drawing equi-density contours. Every EM grid point with density exceeding a selected cutoff value was listed as a virtual atom whose coordinates are those of the grid point and whose radius is √3G/2, where G is the EM grid interval (this representation is similar to the virtual atoms representation of the electrostatic potential by Heifetz et al. [40]). The virtual atoms thus fill the volume delimited by the selected equi-density contour. Each virtual atom is assigned the density value of the related grid point and in this way the selected part of the EM map can be easily rotated and shifted to various positions.

### *Fit*EM2EMin – a tool for matching low resolution 3D structures

The *Fit*EM2EMin algorithm is based on the FFT docking procedure, MolFit, initially described by Katchalski-Katzir and coworkers [23]. The objects to be matched are represented by cubic grids. The center of the grid is at the centroid of the object and grid points are assigned values with regard to their position relative to the object. Grid points within the volume of any virtual atom are considered part of the object (interior or surface); other grid points are assigned “outside the object” values. Distinction between the interior and a surface layer is made for the stationary object A. The resultant grid representations are as follows:

Object A (the stationary object):
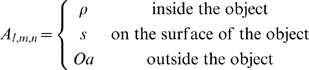



Object B (the moving object):
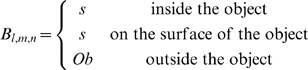



The grid representations are correlated using FFT, producing the matrix C*_α,β,γ_* (eq. 1). C holds the correlation scores for shifts of *B_l,m,n_* with respect to *A_l,m,n_* by *α*, *β*, and *γ* grid points along three perpendicular axes.
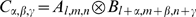
(1)By setting *Oa* to a negative value, *s* to a positive value and *ρ* and *Ob* to zero, the correlation scores are positive when object B is positioned within the volume of object A and overlaps its surface layer. The correlation scores reflect the degree of surface similarity between objects A and B thus higher positive scores indicate more extensive matching of the surfaces and little or no protrusion of object B outside of the volume of object A. The *n* highest scoring *α*, *β*, *γ* shifts in the current correlation matrix are identified and saved.

To complete a six dimensional search in the rotation-translation space the moving object is rotated to a new orientation (by applying a rotation transformation to the coordinates of the virtual atoms), its grid representation is recalculated for the new orientation, and then a new correlation matrix is calculated and new high scoring positions are identified and saved. The procedure is repeated until the stepwise rotational scan is completed and then all the saved models are sorted by their correlation scores.

The surface of object A consists of at least 2 layers of voxels; it was designed such that the inner layer follows the shape of the EM object (at the selected density cutoff) and the outer layer is extended and smoothed. The extension and smoothing are achieved by increasing the virtual atoms radii to (√3/2+*w*)*g*, where *g* is the *Fit*EM2EMin grid interval and *w* is a free parameter. A larger value of *w* produces a more extended and smoother outer surface because small holes and crevices are eliminated. The surface of object B is not distinguished from its interior however the object's volume is extended and the surface is smoothed by setting the virtual atoms radii to (√3/2+1)*g*.

The parameters *s*, *ρ*, *Oa*, *Ob* and *w* determine the shapes of the objects and their grid representations; the grid interval, the rotational grid step and *n* are scanning parameters. Importantly, the grid interval employed in *Fit*EM2EMin is not necessarily the same grid interval as in the EM map; this freedom is made feasible by the virtual atoms representation of the EM maps that allows projection of these spheres onto cubic grids of any size. The values of *ρ* and *s* were selected to be −15 and 1, respectively. These values were previously determined for the high-to-high (atomic) resolution docking program MolFit [23]. The self-matching calibration tests described in the Results section show them to be adequate for low-to-low resolution matching. We chose to use a grid interval of similar magnitude to the grid interval in the experimental EM maps where possible because a smaller grid interval can create artifacts whereas a larger grid interval effectively lowers the resolution of the map. In cases when different experimental EM maps were matched, with different grid intervals, we used the average interval to reduce the error. The rotational grid interval was set to 12°, which was previously found to be adequate for high-to-high resolution docking [41], high-to-low resolution docking [22] and proved adequate in the calibration scans of *Fit*EM2EMin. We show in the results that use of similar rotational intervals does not affect significantly the matching results.

### *Fit*EM2EMout – a tool for docking low resolution 3D structures

By setting *s* to a positive value, *ρ* to a negative value and *Oa* and *Ob* to zero, the correlation score is positive only when object B is positioned outside of and in contact with object A. The correlation score is higher when the contact area (surface overlap) is larger and there is no or very little interpenetration of the objects. The value of *w* optimized for the *Fit*EM2EMin procedure was used also for docking. The *Fit*EM2EMout grid interval was made the same as the grid interval in the EM map.

### DifferenceGrid – a tool for comparing objects

This tool calculates the difference between grid representations of two objects. The virtual atoms radii of both objects are set to √3/2 *g*. Grid points within object A are given the value *s* and the exterior is set to *Oa*; the corresponding grid values for object B are set to *s* and *Ob*, respectively. The difference between the two grid representations is equal to 0 where both objects overlap and to *Oa*-*Ob* where the exterior overlap; it is equal to *Oa*-*s* where object B protrudes out of object A and to *Ob*-*s* where object A protrudes out of object B.
